# Supplementary material for: Waste Citrus reticulata Assisted Preparation of Cobalt Oxide Nanoparticles for Supercapacitors
Source: Nanomaterials (Basel). 2022 Nov 22;12(23):4119. doi: 10.3390/nano12234119 (PMC9739854; doi:10.3390/nano12234119)
Supplement: Supplementary file 1 [file nanomaterials-12-04119-s001.zip › nanomaterials-2043387-supplementary.pdf]

Supporting Information

Waste *Citrus reticulata* Assisted Preparation of Cobalt Oxide Nanoparticles for Supercapacitors

Rishabh Srivastava [1,2], Shiva Bhardwaj [1,2], Anuj Kumar [3,*], Rahul Singhal [4], Jules Scanley [5], Christine C. Broadbridge [5] and Ram K. Gupta [2,6,*]

[1] Department of Physics, Pittsburg State University, Pittsburg, KS 66762, USA
[2] National Institute of Material Advancement, Pittsburg, KS 66762, USA
[3] Nano-Technology Research Laboratory, Department of Chemistry, GLA University, Mathura 281406, Uttar Pradesh, India
[4] Department of Physics and Engineering Physics, Central Connecticut State University, New Britain, CT 06050, USA
[5] Connecticut State Colleges and Universities (CSCU) Center for Nanotechnology, Southern Connecticut State University, New Haven, CT 06515, USA
[6] Department of Chemistry, Pittsburg State University, Pittsburg, KS 66762, USA
* Correspondence: anuj.kumar@gla.ac.in (A.K.); ramguptamsu@gmail.com (R.K.G.)

-

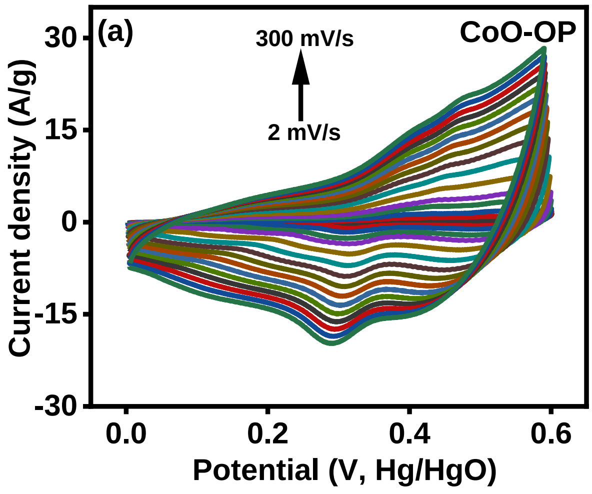

Figure S1: The CV scan curve from 2 to 300 mV/s for prepared (a) CoO-OP.

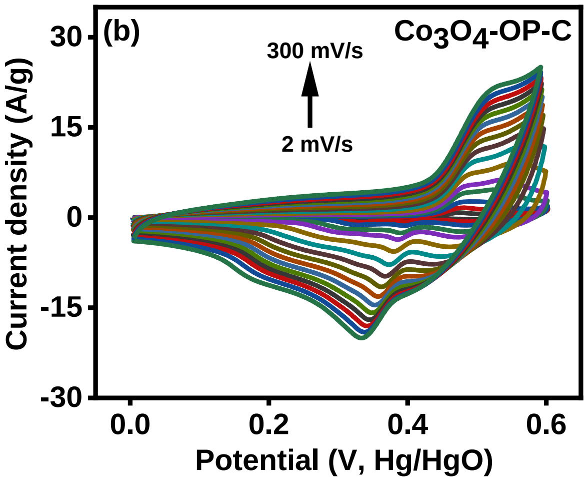

Figure S2: The CV scan curve from 2 to 300 mV/s for prepared (b) Co_3O_4-OP-C.

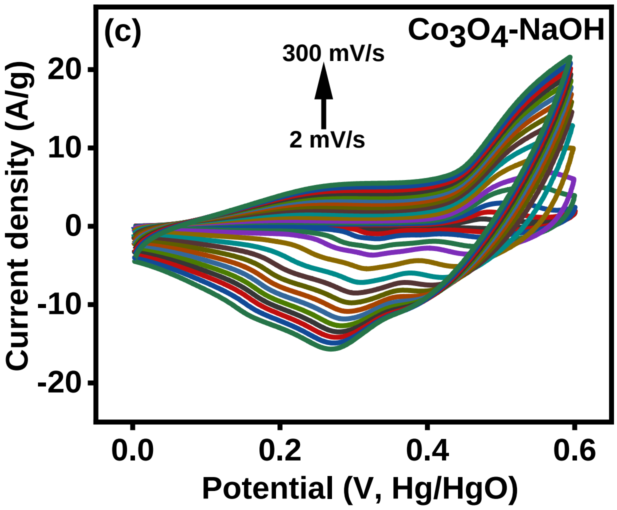

Figure S3: The CV scan curve from 2 to 300 mV/s for prepared (c) Co_3O_4-NaOH.

Table S1: The comparison between specific capacitance (C_{sp}), power density, and energy density of the synthesized samples.

Sample Name	CoO-OP	Co_3O_4-OP-C	Co_3O_4-NaOH	CoO-S-OP	Co_3O_4-S-OP-C	Co_3O_4-S-NaOH	CoO-P-OP	Co_3O_4-P-OP-C	Co_3O_4-P-NaOH
C_{sp} @ 1 A/g	90	53	89	98	87	101	185	100	131
Power density (Wh/Kg)	292	286	284	289	290	294	296	293	293
Energy density (W/Kg)	4.3	2.4	4.0	4.6	4.1	4.8	9.0	4.7	6.2
